# News and Notes

**Published:** 1906-12

**Authors:** 


### NEWS AND NOTES.

NEW ORLEANS is carrying out an order of the board of health to vaccinate all the school-children in the city free.

ANDREW CARNEGIE has given an additional $15,000 to the Atlanta College of Physicians and Surgeons to complete the building fund.

THE New York health board is instituting test experiments in regard to the theory of the opsonic index established by Professor Wright, of London.

DR. C. B. STROTHER, of Albany, Ga., died October 31, at Albuquerque, N. M., where he went a number of years ago for relief from pulmonary tuberculosis.

THE board of public safety, Louisville, on October 18, issued an order requiring physicians of the city to report all cases of tuberculosis to the health office.

THE officers of the Fifth District Medical Society are Dr. J. Olmsted, Atlanta, president; Dr. E. Bates Block, Atlanta, vice-president, and Dr. Elias W. Ragsdale, Covington, secretary and treasurer.

THE German medical press proposes to "blacklist" the authors who contribute "write-ups" for pay and those who publish an article in more than one journal without the knowledge of the publishers.

THE third of the series of Harvey Medical Society lectures was given at the Academy of Medicine building, New York, on November 17, by Professor W. T. Porter, of Boston. His subject was "Vasomotor Reflexes."

---

The Harveian Oration before the Royal College of Physicians, London, was delivered by Dr. William Osler, October 18, 1906, on "The Growth of Truth, as Illustrated in the Discovery of the Circulation of the Blood."

---

A NEW disease, and one which has proved fatal, has appeared among the cattle of Kern county, Cal. It is thought to be due to the cattle feeding on swamps which have lately been under water, but so far no bacteriological investigation has been made.

---

SIR HENRY A. BLAKE, governor of Ceylon, intends to test the efficacy of quinine given at regular intervals as a prophylatic during the malarial season. Malaria is one of the scourges of the island and the trials that have been made lend hopefulness to the experiment.

---

"OLD MARYLAND" is the name of a medical monthly devoted to the interests of the University of Maryland, edited by Dr. Eugene F. Cordell, who desires the support and co-operation of the alumni of that well-known university in making his journal a success and credit to the school.

---

THE nineteenth annual meeting of the Southern Surgical and Gynecological Association will be held in Baltimore December 11, 12 and 13, 1906. The present officers of the association are Dr. George H. Noble, Atlanta, Ga., president; Dr. Stuart McGuire, Richmond, Va., and Dr. E. Denegre Martin,

New Orleans, La., vice-presidents; Dr. Charles M. Rosser, Dallas, Texas, treasurer, and Dr. Howard A. Kelly, Baltimore, Md., chairman committee of arrangements.

---

THE birth-rate of Paris continues to shrink, according to the latest report of the health authorities. There have been fewer births this year than last in every arrondissement except two, including one where the population is growing at the rate of 3,000 annually. One shows 400 fewer births than two years ago.

---

GEO. RASHID, the Syrian leper, whose wanderings several months ago, when he was driven from West Virginia, then from Maryland, then from Pennsylvania, and finally returned to West Virginia by the Maryland authorities, caused much public attention, died recently in a little shanty where he had been imprisoned under guard.

---

THE French senate is now considering a National Old Age Pension Bill. It has been carried through the chamber of deputies with the large majority of 512 to 5. It provides for the payment of $60 a year to all workers, laborers, artisans, clerks, men and women, over sixty years of age. About 2,900,000 persons will come within this scheme.

---

MR. JONATHAN HUTCHINSON, of London, has recently made a visit to Switzerland to ascertain facts in substantiation of his claim that leprosy is caused by eating stale fish. He found that, in the villages in which the disease is present, cured fish of poor quality is imported and eaten in considerable quantities, especially in the winter and spring months.

On November 1, the former and present members of the medical staff of the Johns Hopkins Hospital, tendered a farewell banquet to Dr. Henry M. Hurd, superintendent of the hospital, who was to sail for Europe on the fifth instant. The feature of the occasion was to be the presentation to the board of trustees of the hospital, by Dr. William H. Welch, of a life-size portrait of Dr. Hurd, which will be hung in the library of the hospital.

---

The Alvarenga Prize will be awarded on July 14, 1907, to the competitor sending in the best work on any subject in medicine. The prize amounts to about $180 dollars. All essays must be typewritten and sent in without signature. A motto must be plainly written at the head of the paper and also on the sealed envelope containing the name of the competitor. For further information address the secretary, Dr. Thos. R. Neilson, College of Physicians, Philadelphia, Pa.

---

A commission consisting of Drs. Bashford, King and Igaravidez has been investigating Porto Rican anemia for the past two years. It finds that in ninety-nine per cent. of the cases infection (*Ankylostomum duodenale*, or uncinaria) occurred by way of the skin, as indicated by the "ground itch" on the feet and ankles. For prophylaxis it is advised that wearing shoes should be made compulsory, all infected persons should use a latrine, and all workers in the soil should wash their hands and clean their nails before eating. Eighteen thousand, eight hundred and sixty-five patients were treated with consecutive doses of thymol or beta-naphthol, and the majority were cured.—*Clinical Review*, *November*, *1906*.

### NEWSPAPER ADVERTISING.

A committee of the Philadelphia County Medical Society prepared the following resolutions to be acted on October 11:

"1. *Resolved*, That the publication in the lay press of the description of operations, new treatment and allied medical matters in connection with the name of the physician be condemned by the society, and that the physicians of the society, in order to prevent such publication, make such representations to the superintendents or other officers of the hospital with which they are connected, as will, so far as possible, prevent internes, nurses, employees or friends from giving information to reporters.

"2. *Resolved*, That the society try to prevent reports of the papers read at its meetings from being publicly mentioned unless such publication be conducive to the public welfare. The society shall instruct its secretary or reporter to give to the press a censored report which shall always be anonymous so far as the authorship of the papers is concerned.

"3. *Resolved*, That the society condemn the publication of medical interviews in connection with the names of physicians, excepting medical interviews relating to municipal sanitation and other matters of public welfare.

"4. *Resolved*, That copies of all articles appearing in the press of Philadelphia relating to regular physicians of that city shall be placed in a scrap-book by the assistant secretary for the inspection of members. A member whose name appears therein shall have the privilege of attaching thereto a written explanation.

"5. *Resolved*, That copies of these resolutions be printed in *American Medicine*, *The Journal of the American Medical Association* and the *Weekly Roster*.—*American Medicine*, October, 1906.

